# Two Cases of Myiasis Associated with Malignancies in Patients Living in the Continental United States

**DOI:** 10.7759/cureus.2049

**Published:** 2018-01-10

**Authors:** Anita Lwanga, Michael Anis, Mohamed Ayoubi, Jaya Sharma, Pam Khosla

**Affiliations:** 1 Division of Academic Internal Medicine and Geriatrics, University of Illinois at Chicago; 2 Internal Medicine, Mount Sinai Hospital; 3 Department of Hematology and Oncology, Mount Sinai Hospital

**Keywords:** myiasis, larvae, malignant wound, gynecological cancers, maggot therapy

## Abstract

Myiasis is the infestation of humans with dipterous larvae. Traditionally, myiasis was thought to affect individuals living in tropical regions, however, several cases in temperate zones have been reported. We encountered two patients with histories of malignancies that presented with complaints of myiasis, in Chicago, in the spring and summer of 2016. The first patient, a 54-year-old female with a history of breast cancer, presented with complaints of maggots infesting her postsurgical chest wounds. She was diagnosed with sepsis, cellulitis, and wound myiasis. The second patient, a 63-year-old female with a history of recurrent ovarian cancer, presented with complaints of passing maggots vaginally and seeing worms mixed with her stools. She was diagnosed with internal urogenital myiasis. The first lesson that we learned from these cases is that myiasis can occur in individuals living in any part of the world. Second of all, for patients with accidental myiasis, a sample of the larvae should be sent for analysis to help guide the treatment. Third of all, myiasis has been associated with new or recurrent malignancies, and therefore a biopsy of the affected tissue should be sent for analysis. Finally, we learned that myiasis can serve as a form of tissue debridement; this coinciding benefit should not prevent the treatment of accidental myiasis.

## Introduction

Myiasis, the infestation of humans with dipterous larvae, is derived from the Greek word “muia” for fly [1-3]. It can be classified ecologically or according to the anatomical site of inoculation [1]. Ecologically, myiasis can be obligatory, facultative, or accidental [1]. Anatomically, myiasis may be classified as bloodsucking, cutaneous or cavitary [1]. Cutaneous myiasis includes the subtypes funicular, migratory, and wound myiasis [1, 4]. In cases of cavitary myiasis, the infestation receives the name of the affected area [1]. For example, infestation of the genitourinary tract is called urogenital myiasis.

In the past, myiasis was thought to affect individuals living in tropical and subtropical regions [1-3, 5-8]. Recently, case reports of myiasis affecting individuals living in temperate zones have been published; this is likely due to an increase in international travel and awareness of its occurrence in temperate zones, in the spring and summer seasons [4].

Risk factors for myiasis include low socioeconomic status, poor hygiene, poverty, older age, psychiatric illness, alcoholism, weakness, diabetes, and vascular occlusive disease [1- 2, 4, 8]. Beyond knowledge about the risk factors, there is a paucity of information on epidemiological data on human myiasis, and as a result, its true impact on humans remains unknown [1, 8]. The lack of data on human myiasis is partially explained by the fact that many healthcare professionals feel that it is of minor importance and therefore do not report cases; larvae and dressings are often discarded without careful examination and infestations are treated by the patient’s family, reducing the number of cases seen in medical facilities [1].

## Case presentation

**Figure 1:**
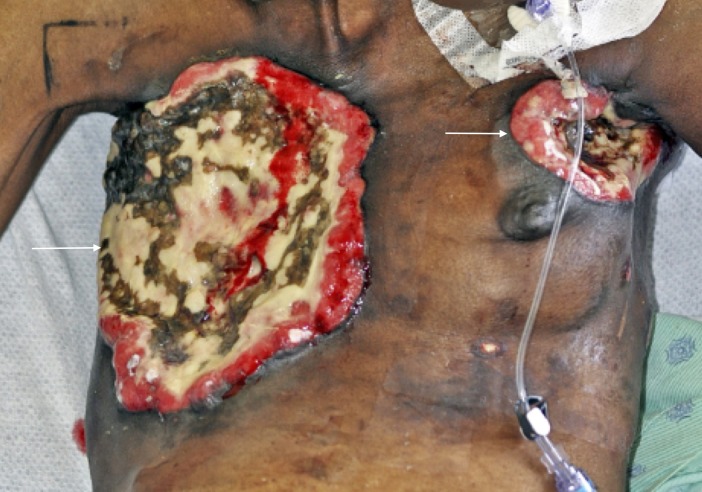
The picture of necrotic lesions affecting the right mastectomy and left lumpectomy sites, two weeks after the treatment of myiasis and cellulitis.

Two patients presented to our institution in the spring and summer of 2016 with complaints of myiasis. Both of the patients had histories of malignancies. The first patient, a 54-year-old female was sent to the hospital for evaluation of chest pain after her mother noticed maggots infiltrating her postsurgical chest wounds. She had a medical history of obsessive-compulsive disorder (OCD) and estrogen receptor (ER) positive, human epidermal receptor 2 (HER 2) negative breast cancer. The breast cancer was initially diagnosed in 2001 in her right breast. The patient received chemotherapy and radiation therapy. In 2013, cancer reoccurred in her right breast and was managed with a right-sided mastectomy. The patient was not able to recall if she received radiation therapy, chemotherapy or hormonal chemotherapy for the reoccurrence. We were unable to obtain medical records pertaining to duration and type of the treatment she received because the hospital where she was initially diagnosed and treated was shut down. In December of 2015, the patient presented to another facility, where she was diagnosed with left-sided breast cancer; she subsequently received a left-sided lumpectomy and attended two sessions of radiation therapy, but she did not complete the treatment because OCD made it difficult for her to attend follow-up appointments. The patient reported that after each of her surgeries, in 2013 and 2015, the wounds at the right and left side of the chest did not heal completely (Figure 1).

At the time of presentation, in 2016, she did not take any medications, have allergies, and did not have any children. Her sister passed away as a result of breast cancer-related complications. The patient was five feet and one inch tall and weighed 86 pounds. Her temperature was 95.7 degrees Fahrenheit, while her heart rate was 103 beats per minute. The rest of her vital signs were within normal limits. Her physical exam was remarkable for a 30 cm x 22 cm necrotic wound covering the right part of her chest. There was another 7 cm x 10 cm necrotic lesion at the left upper breast. Both wounds had maggots burrowing through them. She also had a large fluctuant left axillary mass and lymphadenopathy affecting the left arm.

The patient was diagnosed with wound myiasis, sepsis secondary to cellulitis, and sternal osteomyelitis. She received intravenous fluids, vancomycin, cefepime, and metronidazole. The emergency department staff irrigated her wounds with peroxide and normal saline and then covered her chest with petrolatum and dry dressings. The surgical team recommended conservative management because there was not enough tissue to debride.

**Figure 2:**
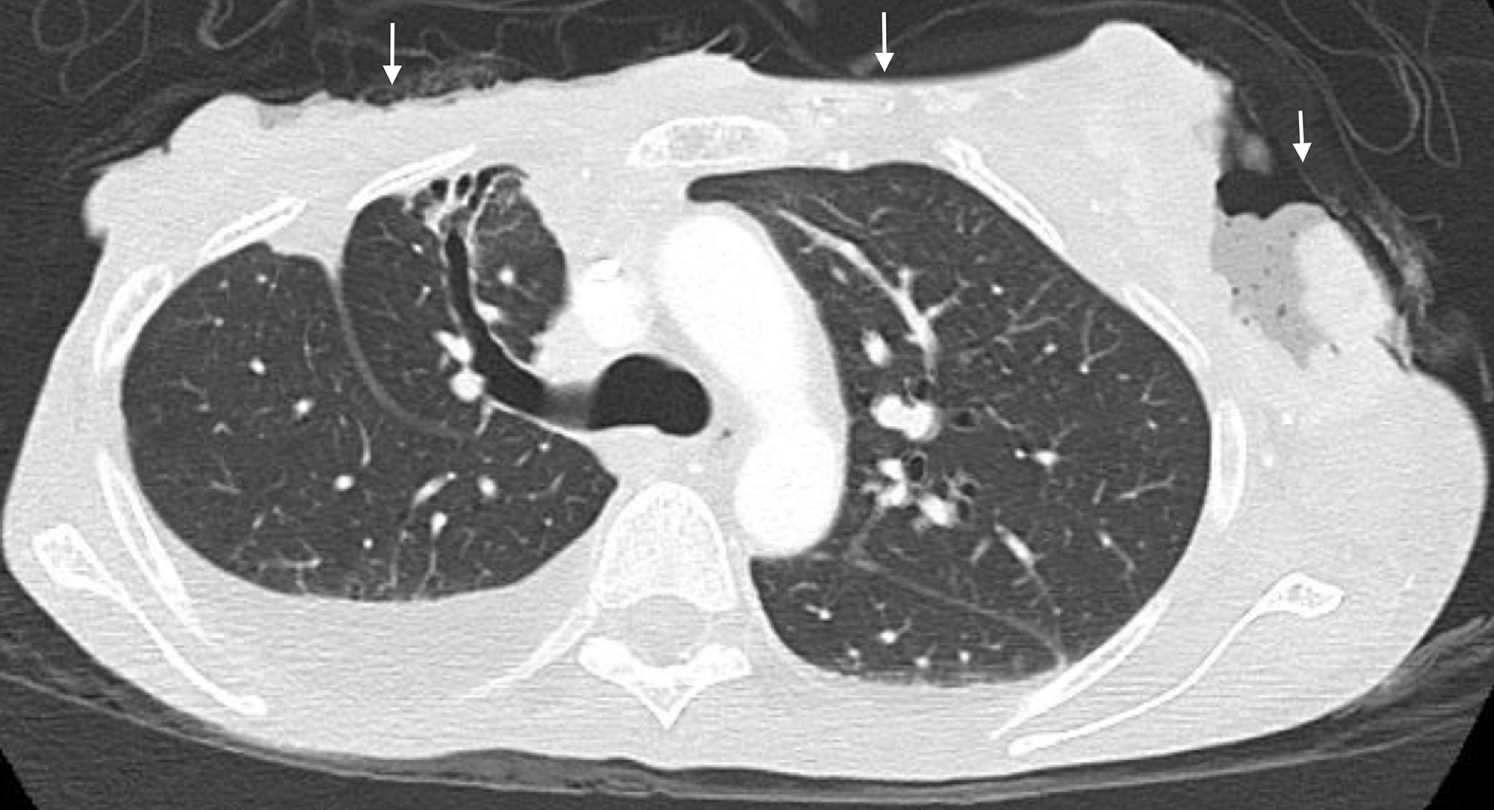
The sagittal cross section from the computed tomography (CT) of the chest that demonstrates the extension of the soft tissue infection at the left lumpectomy and the right mastectomy sites into the sternum and mediastinum.

**Figure 3:**
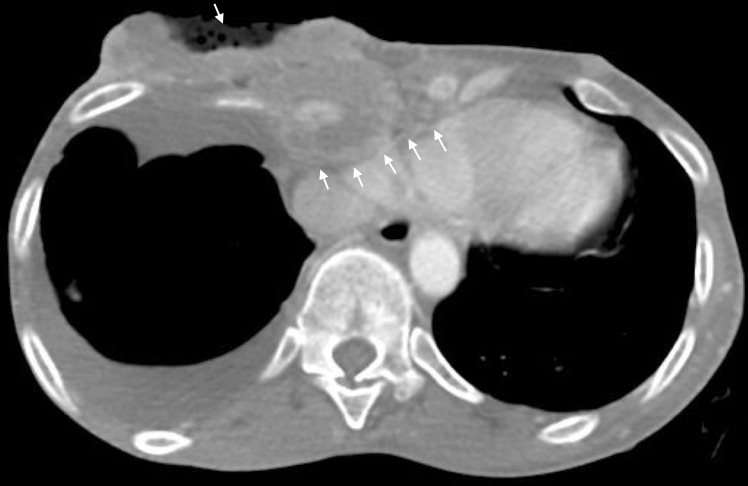
The sagittal cross section from the computed tomography (CT) of the chest that shows the extension of the wounds into the mediastinum.

**Figure 4:**
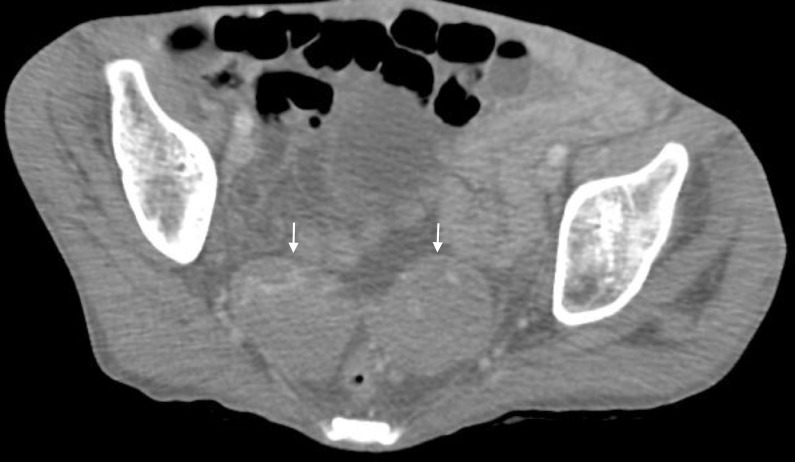
The sagittal cross section from the computed tomography (CT) of the pelvis of the bilateral adnexal masses.

Once the patient was stabilized, a computed tomography (CT) scan of the chest, abdomen, and pelvis was done, which revealed extension of the soft tissue infection into the sternum and mediastinum with mass effect on the left atrium (Figures 2-3). There were metastatic lesions in the lungs. Bilateral adnexal masses were also noted on the CT of the pelvis (Figure 4). The magnetic resonance imaging (MRI) scan of the brain was negative for metastasis.

**Figure 5:**
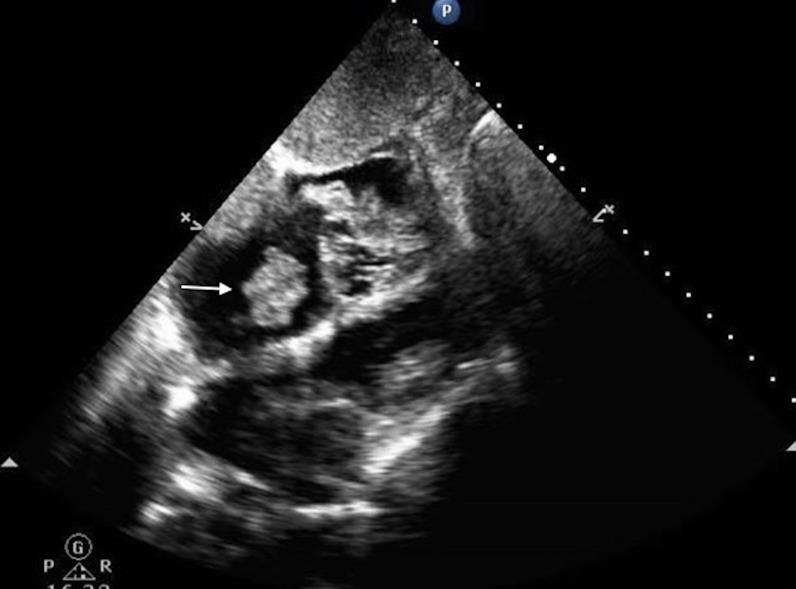
The image from the echocardiogram demonstrating a 2.1 x 2.2 cm thrombus in the right atrium.

The patient's course was further complicated by blood cultures that grew coagulase-negative staphylococcus aureus and moraxella species. An echocardiogram was done, which was negative for vegetations but revealed a 2.1 cm x 2.2 cm thrombus in the right atrium (Figure 5). The patient was started on subcutaneous enoxaparin, rather than warfarin, because she had an increased risk of bleeding while taking warfarin, due to her poor nutritional status. After resolution of the cellulitis, the patient received 3000 centigray (cGY) of palliative radiation therapy, over 10 fractions, to the chest.

The patient was discharged to a nursing home and continued to follow up for chemotherapy. She received paclitaxel, weekly, for seven weeks. A few days after being discharged from the nursing home, the patient passed away. We were unable to obtain information about the cause of her death.

**Figure 6:**
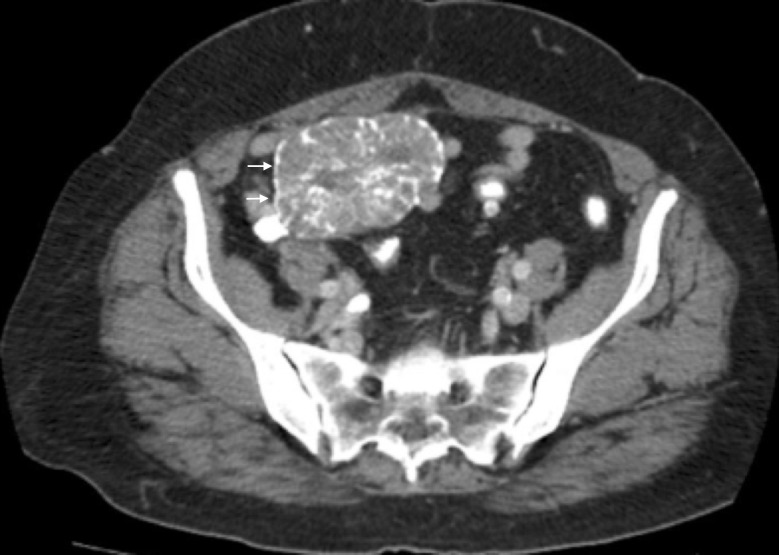
The sagittal cross section from the computed tomography (CT) of the abdomen and pelvis, performed in 2009, illustrating one of the ovarian masses.

**Figure 7:**
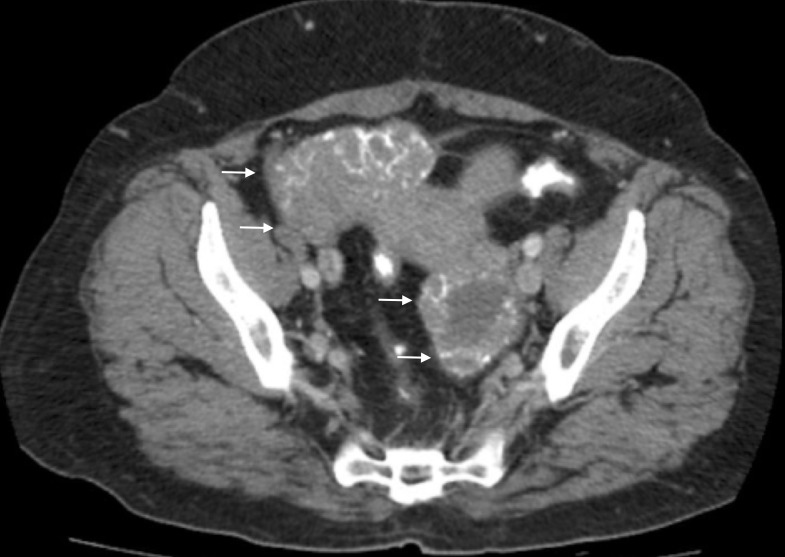
The additional image from the computed tomography (CT) of the abdomen and pelvis, performed in 2009, of the ovarian masses.

The second patient, a 63-year-old female, presented with complaints of passing a 'ball of worms' through her vagina and passing stools mixed with worms. Her review of systems was positive for weakness, weight loss, and blood mixed with her stool. She had a medical history of anemia, bipolar disorder, depression, bilateral estrogen receptor (ER) positive, progesterone receptor (PR) positive, p53 negative, stage IIIC bilateral ovarian psammomacarcinoma. The cancer was initially diagnosed in 2009. At the time, her cancer antigen (CA) 125 was 73 U/mL (normal 0-35 U/mL) and computed tomography (CT) scans of the abdomen and pelvis identified pelvic masses with extensive calcification (Figures 6-7).

The patient received a total abdominal hysterectomy and bilateral salpingo-oophorectomy. Histopathology confirmed the presence of psammoma bodies. She received six cycles of adjuvant carboplatin and paclitaxel but stopped attending follow-up appointments after completing chemotherapy. In 2014, during an elective laparoscopic ventral hernia repair, she was noted to have metastatic lesions in the omentum and peritoneum. The CA 125 was 66 U/mL at that time. The patient was informed of the reoccurrence but did not attend follow-up appointments. In 2015, she presented to her gynecologist with complaints of rectal bleeding. Additional CT scans of the abdomen and pelvis confirmed that the mass had reoccurred and appeared to involve the colon. At the time, her CA 125 increased to 110 U/mL. The patient was referred to a gastroenterologist for a colonoscopy but did not follow up. 

In 2016, when the patient presented with myiasis, she did not take any medications and did not have allergies. She was homeless, consumed alcohol daily, and denied traveling outside of the country. Her family history was non-contributory. The patient was four feet and nine inches tall and weighed 112 pounds. Her vital signs were within normal limits. Larvae were not visualized on external examination of the labia and rectum; the rest of her physical exam was unremarkable. She was re-referred to her gynecologist for pelvic washings.

**Figure 8:**
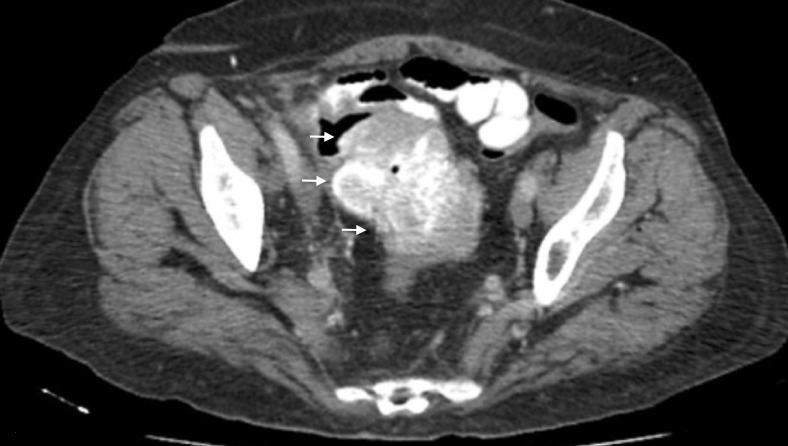
The sagittal cross section from the computed tomography (CT) of the abdomen and pelvis, performed in 2016, of the ovarian mass.

**Figure 9:**
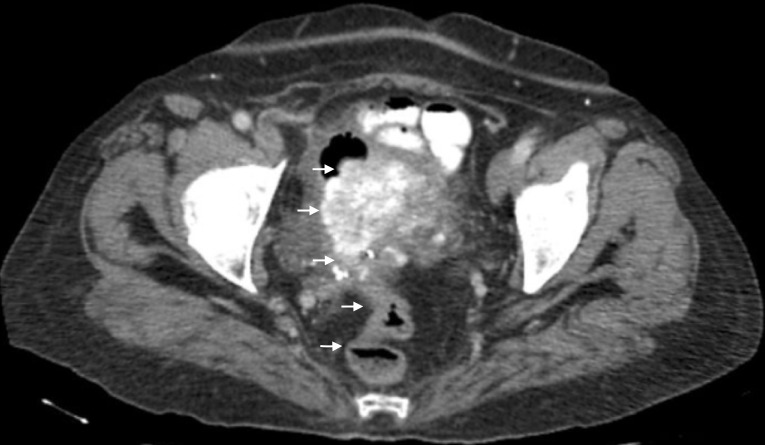
The additional image from the computed tomography (CT) of the abdomen and pelvis, performed in 2016, of the ovarian mass with extension into the colon.

**Figure 10:**
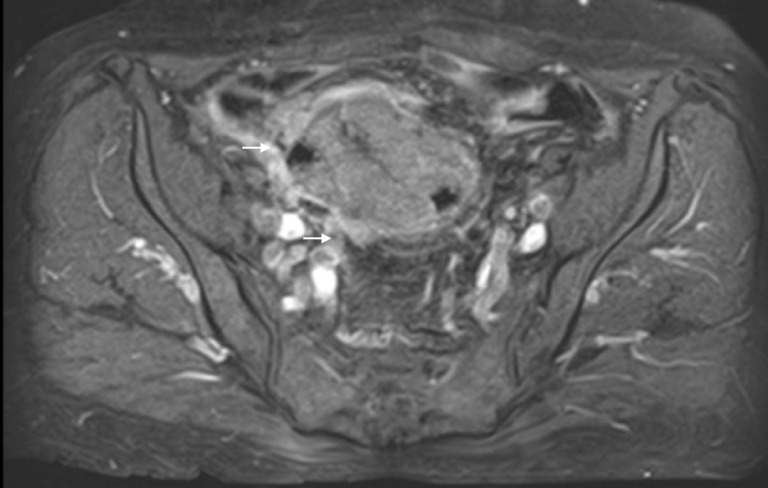
The sagittal cross section from the magnetic resonance imaging (MRI) of the abdomen-pelvis, performed in 2016, of the ovarian mass.

**Figure 11:**
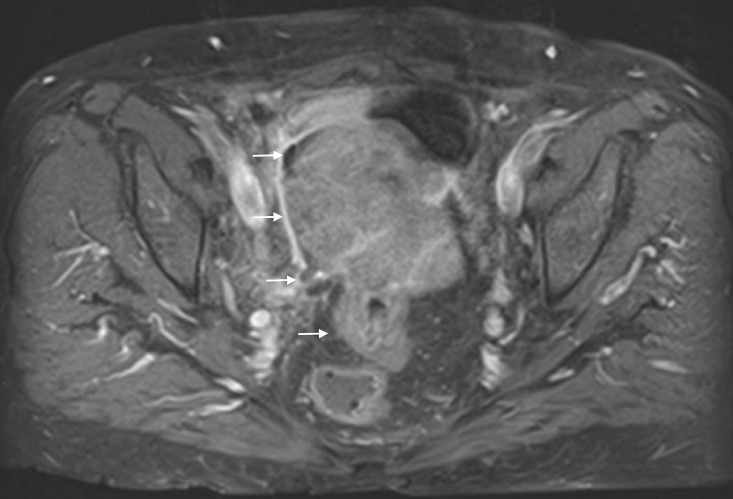
The additional image from the magnetic resonance imaging (MRI) of the abdomen and pelvis, of the ovarian mass, which demonstrates that the mass extends into the colon.

**Figure 12:**
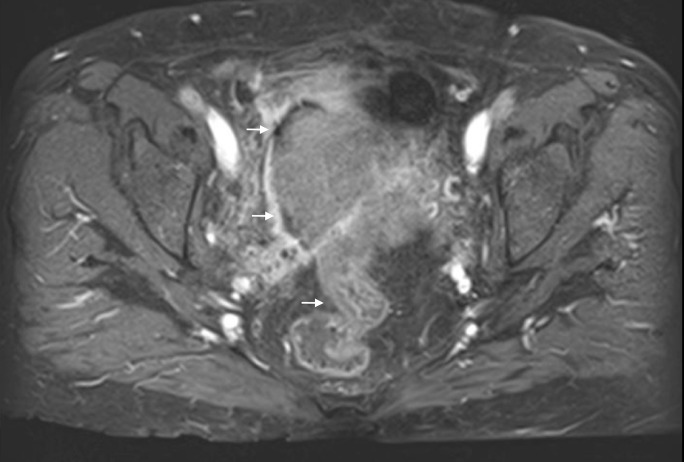
The additional image from the magnetic resonance imaging (MRI) of the abdomen and pelvis demonstrating the abdominal mass.

The patient had additional CT scans of the abdomen and pelvis, which identified a mass in the pelvis that appeared to extend into the small bowel and colon, with fistulization of the mass into the urinary bladder (Figures 8-9). The CT scan of the brain was negative for metastasis. To better characterize the mass, MRI of the abdomen and pelvis was performed, which confirmed that ovarian cancer had spread to the colon and bladder; extensive scarring and post-radiation fibrosis of the bladder and colon were also noted (Figures 10-12). The patient agreed to have a flexible sigmoidoscopy with biopsy, which confirmed the presence of a large ulcerating fungating colonic mass. Histopathology was consistent with micropapillary serous psammocarcinoma with psammoma bodies.

Despite being informed of the findings, the patient continued to decline surgery. She received radiation therapy to the pelvis to help manage the vaginal bleeding, along with carboplatin and paclitaxel. One year after presenting with myiasis, the patient denied seeing additional maggots, the vaginal bleeding had resolved, and she had gained weight. The repeat CT scans of the abdomen and pelvis confirmed that the mass was stable in size when compared to the previous imaging studies.

## Discussion

Both of the patients had several risk factors for myiasis including limited finances and untreated psychiatric illness, which hindered their ability to follow up with their healthcare providers. The second patient had additional risk factors of homelessness and alcoholism.

As discussed in the introduction, health care workers often take myiasis for granted. At our institution surgical residents reported seeing myiasis often, but they did not report the cases because they felt that they were commonplace. When a patient complains of myiasis, a specimen of the larvae should be sent for analysis, to help confirm the diagnosis, determine the appropriate course of treatment, and collect epidemiological data on the incidence of myiasis in humans [1, 4]. Proper identification of dipterous larvae requires the skill of an experienced pathologist, entomologist or parasitologist [4]. Prior to submitting the specimen, information should be obtained about how to kill, and or preserve the specimen, as recommendations may vary between laboratories [4]. Despite presenting with myiasis, neither of the patients had samples sent for analysis. Fortunately, both of the patients had their stories corroborated by their health care providers and family members who saw the maggots. 

The first patient had malignant wound myiasis. In some cases, myiasis may herald a malignancy or reoccurrence; this occurs because malignant wounds exude volatile metabolites, blood, and decaying tissues, which attract flies [9]. Patients presenting with wound myiasis should have a biopsy of the affected tissue to determine if there is an underlying malignancy [9]. If a malignancy is confirmed, the patient should receive tetanus prophylaxis and surgical excision [1, 9]. If surgery is not feasible, irrigation with saline and antiseptic solutions, application of viscous substances to suffocate the larvae, mechanical removal, and chemical debridement can be done [1, 4]. In cases where mechanical removal is not necessary, the patient may be treated with a larvicide such as oral Ivermectin [1]. The first patient received local wound therapy and antibiotics. A biopsy of the wounds was not performed because the patient had a history of partially treated breast cancer and non-healing surgical wounds.

In the first patient's case, the surgical team did not find enough dead tissue to debride. Accidental myiasis may have been a blessing in disguise because the maggots likely consumed the majority of necrotic tissue. Maggot therapy, which is controlled and artificially induced myiasis, was developed from observational studies of the benefits of maggot infestation on the wounds of injured soldiers [4]. The larvae perform debridement, produce bactericidal substances that promote granulation, and reduce the risk of a bacterial co-infection [1, 10]. The benefits of maggot therapy should not preclude treatment of accidental myiasis because an uncontrolled infestation can cause extensive tissue damage and traumatize the patient.

The second patient had internal urogenital myiasis, which is an uncommon subtype of accidental myiasis [1]. The symptoms of flank pain, and abdominal pain usually subside after expelling or removal of the maggots [1]. In most cases, the diagnosis of internal urogenital myiasis is made after the maggots have already been expelled [1], which was the case with this patient.

## Conclusions

Several lessons can be learned from these cases. First of all, physicians should be aware that myiasis can occur in individuals living in any part of the world. Second of all, when a patient complains of myiasis, samples of the larvae should be sent for analysis to confirm the diagnosis, guide treatment with larvicides, and help collect epidemiological data on the incidence of myiasis in humans. Third of all, in cases of wound myiasis, a biopsy of the wound may be done to rule out a new malignancy or a reoccurrence. Finally, wound myiasis, whether accidental or iatrogenic, can serve as a form of tissue debridement and deter the growth of a malignancy, but these benefits should not prevent the treatment of accidental myiasis.
